# Near infrared light controlled gene editing

**DOI:** 10.1038/s41377-025-02128-x

**Published:** 2026-01-05

**Authors:** Mikhail Y. Berezin

**Affiliations:** Washington University School of Medicine, St. Louis, MO 63110 USA

**Keywords:** Optics and photonics, Other photonics

## Abstract

A novel NIR light-activated CRISPR-dCas9/Cas9 system achieves precise and rapid gene regulation in living organism using a chemically cleavable rapamycin dimer. Unlike previous light-driven systems, this approach offers deeper tissue penetration, low toxicity, fast response, and minimal background activity. This platform opens new directions for highly efficient, targeted, noninvasive, and spatially confined gene editing for a great number of preclinical and clinically translatable applications.

CRISPR (Clustered Regularly Interspaced Short Palindromic Repeats) technology for studying gene function and uncovering mechanisms^1^, in the hands of biologists, has fundamentally reshaped modern biology. This method enabled the creation of thousands of cells and animal models with precisely engineered features. The work that once required years of trial-and-error experiments now, with CRISPR, can be performed within weeks rapidly and reproducibly, transforming the pace of discovery in biology and promising to change the future medicine.

But in the hands of biomedical engineers and imaging scientists, CRISPR takes on a new height. It is no longer just a tool for editing the genome, but also a part of the optical toolbox, together with other optical instruments such as lasers, lenses, and detectors. This new tool adds another dimension by enabling researchers to control gene expression and its functions by optical means through the tissue. This expanded toolbox is especially powerful when applied to small animals, where the longstanding challenge of inducing these changes non-invasively deep inside of living animals, in an exact location and in real time, has long been considered the “holy grail” of modern biology.

The study by Zhang et al.^2^, led by the scientists from the Center for Advanced Biomedical Imaging and Photonics at Harvard University and RNA Institute from the University at Albany recently published in *Light: Science & Applications*, addresses this high-bar challenge by introducing a CRISPR system that can be activated deep within tissues using light. Their innovative platform combines clever synthetic chemistry, gene editing, and optical engineering to produce a system that not only edits genes but does so on demand, with light, and with unprecedented spatial control.

Photoactivatable CRISPR systems, which only function upon illumination, have shown great promise in a number of cell studies over the last decade^3–5^. These systems have been shown to achieve robust OFF-ON switching with minimal background activity, rapid activation upon light exposure, and high editing efficiency in mammalian cells, as well as in model organisms such as zebrafish embryos^6^. Efforts to regulate CRISPR activity using light have taken many creative forms. Blue-light-inducible systems, such as those employing CRY2-CIB1 or Magnet-based heterodimerization domains^3^, allow for reversible control of Cas9 or dCas9 activity. Other strategies rely on caged guide RNAs or photocaged amino acids to block CRISPR function until uncaged by ultraviolet (UV) light^6,7^. However, these technologies face significant translational obstacles.

Most of such systems rely on UV or blue light, which suffer from limited tissue penetration due to the high scattering of these high energy photons by the tissue (recall why sky is blue). There are also the concerns about high absorption of these photons by tissue components, and phototoxicity resulting in producing strong radicals and damaging DNA (recall why we need sunscreen). These drawbacks make the use photoactivatable CRISPR systems in any in vivo applications impractical. To overcome these issues, Zhang et al. introduce an elegant solution based on a near-infrared (NIR) light-activatable CRISPR system.

NIR photons can travel relatively deep into the tissue without much scattering or being absorbed by the tissue components (i.e. blood, and other tissues are relatively transparent in the NIR range) and their energy is too low to cause unwanted biochemical reactions. For that reason, NIR is widely used approach in the imaging of small animals^8,9^, but not that much in gene editing.

The idea to move to the NIR range has been long attractive. However, close to the NIR range (red and far-red-activated) systems that have been developed in recent years were hampered by slow activation kinetics^7,10^. Zhang et al. solve these challenges with a unique two step approach illustrated by Fig. 1.

**Fig. 1:**
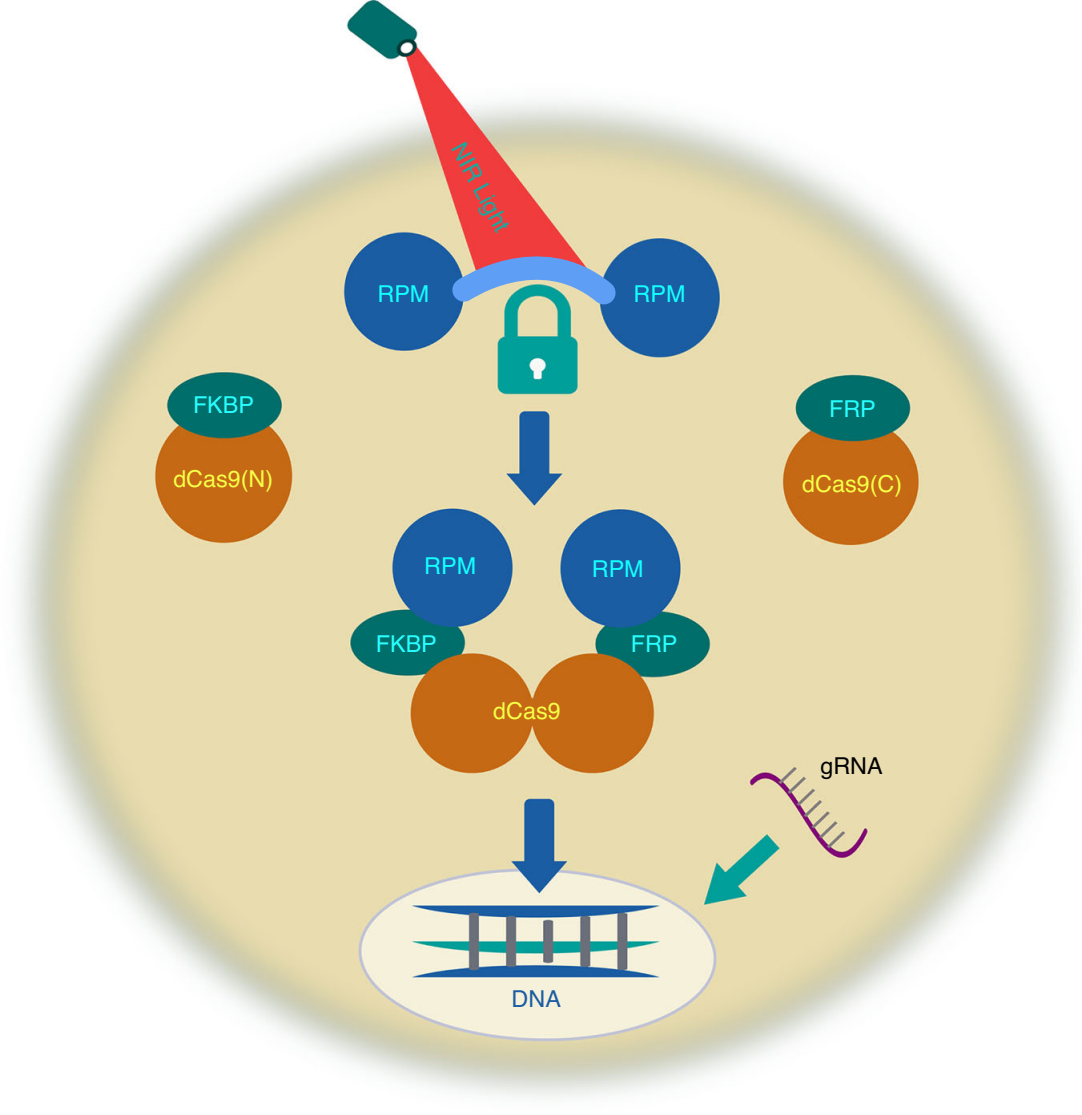
**Schematic of the NIR light activatable chemically induced CRISPR system: NIR light → rapamycin release** **→** **CRISPR reconstitution.** Upon NIR light exposure, the IR780 dye—covalently linked to rapamycin (RPM) dimers - is photocleaved, releasing free RPM molecules. These released RPMs bind to FKBP and FRB domains that are fused to the N- and C-terminal halves of split dCas9 proteins. The resulting dimerization of dCas9(N) and dCas9(C) reconstitutes a functional dCas9 complex. Guided by a specific gRNA, the reassembled dCas9 is directed to the target genomic site, enabling gene modulation

The approach developed represents a paradigm shift in CRISPR control: it introduces a two-component activation system that merges light-triggered chemistry with conditional protein assembly. At its core is a specially engineered rapamycin dimer linked via a photocleavable NIR dye, which makes the contract biologically inert until exposed to light. The usage of the dye is itself an innovation. Most of the efforts were to design NIR dye photostable, weak photostability has been considered to be detrimental in bioimaging applications. The authors of the paper exploit dye’s vulnerability to photobleaching as an asset. Upon irradiation, IR780 (extensively chemically modified to bind rapamycin) undergoes a regioselective photooxidative cleavage that disrupts the caged structure, rapidly freeing rapamycin in its active form. The released rapamycin retained its ability to reconstitute split Cas9/dCas9 with high efficiency, while the overall construct remained stable, cell-permeable, and biocompatible. This reaction occurs within seconds and is robust under biologically relevant conditions. This highly creative molecular engineering feat enabled rapid, reversible, and highly specific gene activation using light.

Looking into the future, the chemically induced, NIR-responsive CRISPR system developed by Zhang et al. offers a powerful new tool for fast and reproducible genome engineering in vivo. While this method has not been applied to the live animals yet, the creative combination of photochemistry with synthetic chemistry and biology, brings this platform closer to the long-sought goal of safe gene therapy in humans.
